# RNF168 facilitates oestrogen receptor ɑ transcription and drives breast cancer proliferation

**DOI:** 10.1111/jcmm.13694

**Published:** 2018-07-05

**Authors:** Zhenhua Liu, Jinghang Zhang, Juntao Xu, Huijie Yang, Xin Li, Yingxiang Hou, Yan Zhao, Min Xue, Beibei Wang, Na Yu, Sifan Yu, Gang Niu, Gaosong Wu, Xiumin Li, Hui Wang, Jian Zhu, Ting Zhuang

**Affiliations:** ^1^ Laboratory of Molecular Oncology Henan Collaborative Innovation Center of Molecular Diagnosis and Laboratory Medicine School of laboratory Medicine Xinxiang Medical University Xinxiang China; ^2^ Henan Key Laboratory of immunology and targeted therapy Xinxiang Medical University Xinxiang China; ^3^ Institute of Lung and Molecular Therapy (ILMT) Xinxiang Medical University Xinxiang China; ^4^ Synthetic Biology Engineering Lab of Henan Province College of Life Science and Technology Xinxiang Medical University Xinxiang China; ^5^ Department of Pathology The First Affiliated Hospital of Xinxiang Medical University Weihui China; ^6^ Rhil Rivers Technology (Beijing) Ltd. Beijing China; ^7^ Department of Cancer Genomics LemonData Biotech (Shenzhen) Shenzhen China; ^8^ Center for Cancer Research Xinxiang Medical University Xinxiang China; ^9^ Key Laboratory of Carcinogenesis and Translational Research (Ministry of Education) Department of Renal cancer and Melanoma Beijing Cancer Hospital and Institute Peking University School of Oncology Beijing China; ^10^ Department of Thyroid and Breast Surgery Zhongnan Hospital Wuhan University Wuhan China; ^11^ Department of Molecular Biology University of Texas Southwestern Medical Center Dallas TX USA

**Keywords:** breast cancer, ER ɑ, RNF168, transcription

## Abstract

Oestrogen receptor ɑ (ERɑ) is overexpressed in two‐thirds of all breast cancers and involves in development and breast cancer progression. Although ERɑ‐positive breast cancer could be effective treated by endocrine therapy, the endocrine resistance is still an urgent clinical problem. Thus, further understanding of the underlying mechanisms ERɑ signalling is critical in dealing with endocrine resistance in breast cancer patients. MCF‐7 and T47D breast cancer cell lines are used to carry out the molecular biological experiments. Western blot is used to assess the relative protein level of ERɑ, RNF168 and actin. Real‐time PCR is used the measure the relative ERɑ‐related gene mRNA level. Luciferase assay is used to measure the relative ERɑ signalling activity. Chromatin immunoprecipitation is used to measure the RNF168 binding affinity to ERɑ promoter regions. WST assay and flow cytometry are used to measure the cell proliferation capacity. We use Student's *t* test and one‐way ANOVA test for statistical data analysis. Here, we report an important role in ERɑ‐positive breast cancer cells for RNF168 protein in supporting cell proliferation by driving the transcription of ERɑ. RNF168 is highly expressed in breast cancer samples, compared with normal breast tissue. In patients with breast cancer, RNF168 expression level is correlated with poor endocrine treatment outcome. Depletion of RNF168 causes decreased cell proliferation in MCF‐7 and T47D cells. Besides, depletion RNF168 reduced mRNA level of ERɑ and its target genes, such as PS2 and GREB1. Chromatin immunoprecipitation revealed that ERɑ transcription is associated with RNF168 recruitment to ERɑ promoter region, suggesting that transcriptional regulation is one mechanism by which RNF168 regulates ERɑ mRNA level and ERɑ signalling in breast cancer cells. RNF168 is required for ERɑ‐positive breast cancer cell proliferation and facilitate ERɑ signalling activity possibly through promoting transcription of ERɑ.

## 1 INTRODUCTION

Oestrogens promote mammary epithelial cell growth in an oestrogen‐dependent manner by stimulating the oestrogen‐inducible genes.^1^ The biological function of estrogens is mainly mediated by binding to oestrogen receptors (ERɑ and ERβ). As the role of ERβ in breast cancer is controversial, ERɑ has been proved to have a main role in breast cancer initiation and proliferation.^2^ Overexpression of ERɑ promotes breast cancer cell growth and correlates with increased oncogenic proteins, including cyclin D1 and c‐myc.^3^ These factors promote cell cycle progression by decreasing the association between cyclin E/CDK2 and CDK inhibitors including P21^Cip1/WAF1^ and P27^kip1^ 4 In clinics, ERɑ levels in dysplastic patients are correlated with the risk of breast cancer, and two‐thirds of all breast cancers maintain high level of ERɑ.^5^, ^6^ Based on the relationship between ERɑ signalling and breast cancer, the subsequent clinical application of anti‐oestrogens brought significant benefits of patients with ERɑ‐positive breast cancer. However, about half of the patients treated by endocrine therapy will eventually relapse, which makes it a significant clinical problem.^7^ Thus, further understanding of the underlying mechanisms and insights into new components of ERɑ signalling is critical in dealing with endocrine resistance in patients with breast cancer.

Among the hundreds of putative E3 in humans, the really interesting new gene (RING) finger protein family has attracted the research attention due the uncommon ubiquitination mechanisms and involvement in chromatin modulations.^8^ Interestingly, quite a few RING finger proteins were proved to involve in modulation ERɑ signalling, such as RNF31, RBCK1, BRCA1 and RNF8.^9^, ^10^, ^11^, ^12^ Some of them may modulate ERɑ protein stability via certain ubiquitination manner, such as RNF31 and RNF8.^9^, ^12^ The others may control ERɑ transcription level, For example, RBCK1 recruitment to ERɑ promoter regions is required for ERɑ gene expression and breast cancer cell proliferation.^10^

RNF168 (RING finger protein 168) was first identified as a novel ubiquitin binding domain (UBD) protein, containing a RING finger motif.^13^ Further studies demonstrated that RNF168 plays an essential role of ubiquitination in DNA damage response (DDR).^14^, ^15^, ^16^ In several DNA repair mechanisms, RNF168 is recruited to DNA damage foci and promotes mono‐ubiquitination of H2A/H2AX at K13‐15 to drive DNA repair process.^14^ Previous studies showed that RNF168 could mediate chemotherapy resistance in several cancer types.^17^ The unbiased public available data show RNF168 is higher expressed in breast cancers compared with normal breast tissue and correlates with poor endocrine treatment outcome.^18^, ^19^ This study identifies the involvement of RNF168 in facilitating ERɑ signalling in breast cancer cells.

## 2 MATERIALS AND METHODS

### 2.1 Cell culture

MCF‐7 cell was cultured in DMEM (Invitrogen, Carlsbad, CA) supplemented with 10% foetal bovine serum (FBS) and 1% penicillin/streptomycin (Invitrogen) at 37°C in a humidified atmosphere of 5% CO2 in air. T47D cells were cultured in RPMI 1640 (Invitrogen) supplemented with 10% FBS (GIBCO) and 1% penicillin/streptomycin. SKBR3 and MDAMB231 cells were cultured in RPMI 1640 (Invitrogen) supplemented with 10% FBS (GIBCO) and 1% penicillin/streptomycin.

### 2.2 siRNA and transfection

Cells were transfected with 50 nmol/L siRNA. RNF168 siRNAs sequences were shown here: RNF168 siRNA #1: 5‐CACAAAGCAUCCAACACCAdTdT‐3; siRNA #2: 5‐GAAGAUAUGCCGACACUUUdTdT‐3. Control siRNA sequences were shown: UUCUCCGAACGUGUCACGUTT. INTERFERin transfection reagent (Polyplus Transfection, 409‐10) was used according to the manufacturer's protocol. Plasmids were transfected by Lipofectamine 2000 (1662298, Invitrogen). The ERE‐TK‐luc reporter and the pRL‐TK control were described in previous study.^20^

### 2.3 RNA extraction and qPCR analysis

RNeasy kits were used to extract total RNA (Qiagen). qPCR was performed as previously described.^21^ 36B4 was used as internal control. Primer sequences for qPCR are provided in Table 1.

**Table 1:** Primer for Q‐PCR

Primer for Q‐PCR	
RNF168 F	5‐ggc gag ttt atg ctg tec ct‐3
RNF168 R	5‐gcc gec acc ttg ctt att tc‐3
GREB1 F	5‐cgt gtg gtg act gga gta gc‐3
GREB1 R	5‐acc tct tea aag cgt gtc gt‐3
PS2 F	5‐cat cga cgt ccc tec aga aga g‐3
PS2 R	5‐ctc tgg gac taa tea ccg tgc tg‐3
PDZK1 F	5‐gcc agg etc. att cat caa aga‐3
PDZK1 R	5‐cct eta gee cag cca agt ca‐3
ESR1 F	5‐gct acg aag tgg gaa tga tga aag‐3
ESR1 R	5‐tct ggc get tgt gtt tea ac‐3
36B4 F	5‐ggc gac ctg gaa gtc caa ct‐3
36B4 R	5‐cca tea gca cca cag cct tc‐3
Primers for ChiP assay	
ESR1 promoter A F	5‐GGG ATC GCT CCA AAT CGA‐3
ESR1 promoter A R	5‐CTT GCC CTG ACA TTG GCT TAA‐3
ESR1 promoter B F	5‐TCA GAT GCC CCC TGT CAG TT‐3
ESR1 promoter B R	5‐CAG CCA GCC ACA GAC AGC TA‐3
ESR1 promoter E2 F	5‐CAG CCC AGC CAA CAT GGT‐3
ESR1 promoter E2 R	5‐GCC CGC CAG CTA ATT TTT TA‐3

### 2.4 Quantification of cell viability

MCF‐7 and T47D cells were transfected with siRNF168 or siControl in 24‐well plates. After 24 hours, the cells were seeded into 96‐well plates. Cell numbers were determined using the WST‐1 cell proliferation reagent as previously described.^22^

### 2.5 Flow cytometry

For ethynyl‐deoxyuridine (EdU) labelled DNA stain, cells were transfected with siRNF168 and siControl. After 24 hours, 10 nmol/L estradiol or vehicle was added for another 24 hours. Then 10 μmol/LEdU was added to each plate for the last 60 minutes. The BD LSR II flow cytometer (BD Bioscience) was used to measure the flow fluorescence intensity.

### 2.6 Western blotting

Cells were lysed with RIPA lysis buffer. Anti‐ERɑ mouse (1D5, SC56833) was from Santa Cruz Biotechnology. Anti‐ERɑ rabbit (D8H8, #8644) was from Cell Signaling Technology. Anti‐RNF168 (SC‐101125) was acquired from Santa Cruz Biotechnology. Anti‐SRC1 (128E7), anti‐SRC3 (5E11) and anti‐H3K27ac (D5E4) antibodies were acquired from Cell Signaling Technology. Anti‐PolII (PLA0127) and anti‐P300 (HPA003128) were acquired from Sigma. Anti‐tubulin (T‐5168) and anti‐histone‐3 (Ab18521) were acquired from Sigma and Abcam, respectively. Anti‐actin (8H10D10) was acquired from Cell Signaling Technology.

### 2.7 Luciferase assay

The luciferase activity was performed using the Dual‐Luciferase Reporter kit (Promega, Germany). The ERE‐luciferase reporter was transfected together with renilla plasmid into the cells. The luciferase activity was measured after 24 hours.

### 2.8 Chromatin immunoprecipitation (ChIP) assay

ChIP assay was performed in our previous study. MCF‐7 cells were fixed for cross‐linking for 30 minutes. After that, the cells were mixed with 0.1375 mol/L glycine, washed with cold PBS/1 mmol/L PMSF and scratched into PBS/1 mmol/L PMSF for centrifuge. Then cells were treated by SDS lysis buffer and sonicated for 10 minutes (30 seconds on/off). Then the ChIP assay kit (Millipore, 17‐295) was used for following steps. The following antibodies were used in the ChIP experiments: anti‐RNF168 (SC‐101125), anti‐SRC1 (128E7), anti‐SRC3 (5E11), anti‐H3K27ac (D5E4), Anti‐PolII (PLA0127) and anti‐P300 (HPA003128) and anti‐ERɑ rabbit (D8H8, #8644). The primer sequences for ChIP assay were shown in Table 1.

### 2.9 RNA sequence analysis

The global gene expression analysis was based on RNA sequencing platform from BGI (Beijing Genomic Institute). The RNA sequence data are deposited in the Gene Expression Omnibus (GEO) database (Assessing number: GSE106617). Analysis was performed for differentially expressed genes (*P* < .01 and fold change >2) by Ingenuity Pathway Analysis (IPA).

### 2.10 Statistics

Student's *t* test and Pearson correlation coefficient were used for comparisons. A *P*‐value of < .05 was considered to be significant.

## 3 RESULTS

### 3.1 RNF168 is higher expressed in breast cancer and correlates with poor prognosis in endocrine treated patients with breast cancer

**Figure 1:**
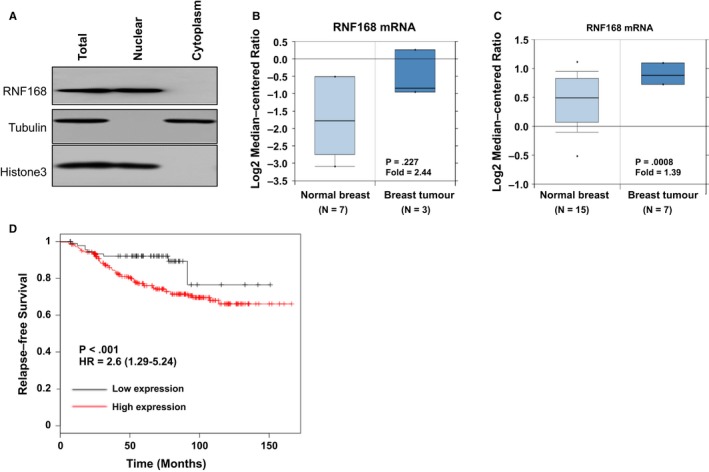
RNF168 is higher expressed in breast cancer and correlates with poor prognosis in endocrine treated patients with breast cancer. A, RNF168 is mainly localized in the nuclear. The subcellular protein fractionation kit (Thermo scientific, 78840) was used for cytoplasm and nuclear separation. Tubulin and histone‐3 were used for cytoplasm and nuclear control. B and C, RNF168 gene expression is higher in breast tumours compared with normal breast tissue. The oncomine database was used to extract the gene expression data (http://oncomine.org). The original gene expression data were from the cited studies.^18^, ^19^ D, RNF168 mRNA level is correlated with poor endocrine treatment outcome in breast cancer patients. The clinical data were acquired from KMPLOT database (http://kmplot.com/analysis/) with the probe ID (226832_at)

We first identify the localization of RNF168 in breast cancer cell line. The cytoplasmic and nuclear separation assay show that RNF168 is mainly located in the nuclear in MCF‐7 cells (Figure 1A). The public available microarray data^18^, ^19^ show that RNF168 is higher expressed in breast cancer samples, compared with normal breast tissues in two independent whole transcriptomic‐based cohorts (Figure 1B,C). Through analysis of the public available breast cancer survival data (http://kmplot.com/analysis/), we observe that RNF168 mRNA level is correlated with poor relapse‐free survival in endocrine treated patients, but not correlated with ER alpha mRNA level (Figures 1D and S1A,B), which might indicate the involvement of RNF168 in regulating oestrogen signalling pathway.

### 3.2 RNF168 depletion inhibits ERɑ‐positive breast cancer cell growth

**Figure 2:**
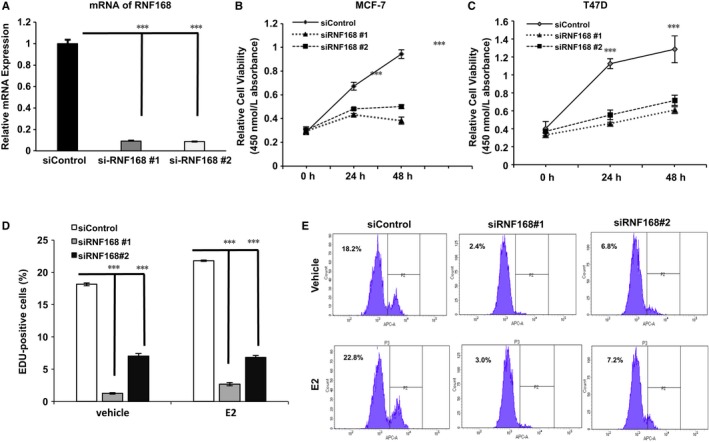
RNF168 depletion inhibits ERɑ‐positive breast cancer cell growth. A, RNF168 depletion effect by two different siRNA oligos. MCF‐7 cell was transfected with siRNF168 or siControl. After 48 h, RNF168 mRNA levels are determined by real‐time PCR with 36B4 as internal control. B and C, The WST‐1 assay was used to determine the cellular metabolic activity at indicated time points after transfection. MCF‐7 and T47D cells were transfected with siRNF168 and siControl. After 24 h, cells were seeded into 96‐well plates. These experiments were performed in triplicates. All values are mean ± SD (n = 3, *P <* *.05; **P < .01, ***P < .001). D and E, RNF168 knockdown decreases cell proliferation in breast cancer cells as determined by EdU incorporation. MCF7 cells were transfected with siRNF168 and siControl. Cells were treated with or without estradiol. EdU was added at a concentration of 10 μmol/L and incubated for 1 h. The cells were subject to FACS analysis. All values are mean ± SD (n = 3, *P <* *.05; **P < .01, ***P < .001). E, showed the representative FACS histogram for EdU incorporation assay

To confirm RNF168 function in ERɑ‐positive breast cancer, we deplete RNF168 expression by two different siRNAs (Figure 2A). RNF168 depletion significantly inhibits cell proliferation in both MCF‐7 and T47D cells by WST‐1 assay (Figure 2B,C). Beside, RNF168 depletion also decreases cell proliferation in SKBR3 and MDAMB231 cells (Figure S2B,C). Further ethynyl‐deoxyuridine (EdU) staining coupled with flow cytometry assay shows that RNF168 depletion dramatically decreases the population of DNA replication cells in both vehicle and estradiol‐treated conditions (Figure 2D,E). Interestingly, estradiol could increase the EdU‐positive cells in control group, but not in the siRNF168 group.

### 3.3 RNF168 depletion decreases ERɑ mRNA and protein level in breast cancer cells

**Figure 3:**
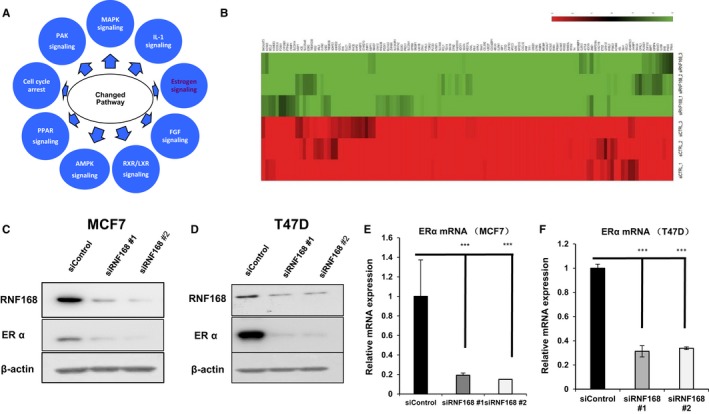
RNF168 depletion decreases ERɑ mRNA and protein level in breast cancer cells. A, Top 10 signalling pathways significantly decreased by RNF168 depletion in MCF7 cells. The pathway‐enrichment analysis was used by the threshold *P* < .001 and fold change >2 to derive regulated genes. SMURF1 was depleted by siRNA (mix of siRNF168 #1 and siRNF168 #2) or treated with siControl. After 48 h, the whole mRNA was extracted for RNA sequence analysis. The siControl and siRNF168 were performed in triplicates. B, The heat‐map graph shows the ERα regulating genes, which is significantly decreased by RNF168 depletion in MCF‐7 cells. The significantly regulated genes were overlapped with publish ERα target gene data.^34^ C and D, RNF168 depletion effect on ERα protein level by two different siRNA oligos. MCF‐7 or T47D cells were transfected with siRNF168 or siControl. After 48 h, RNF168 and ERα protein levels were determined by Western blot analysis. Actin was used as internal control. E and F, RNF168 depletion decreases ERα gene expression using two different siRNA oligos. MCF‐7 and T47D cells were transfected with siRNF168 or siControl. After 48 h, total RNA was prepared and the expression of the endogenous ERα mRNA level by qPCR. Shown are the results from three experiments. *P < .05; **P < .01; ***P < .001 for gene expression comparison

To approach the function of RNF168 in breast cancer cells in an unbiased way, we carried out the whole transcriptomic‐based RNA sequence by comparison between control and RNF168 depletion in MFC‐7 cells. We set *P* value < .001 as the significance threshold. By comparison with siControl group, RNF168 depletion enriches 5529 significantly changed genes, which are associated with several biological processes and several signalling pathways. The pathway‐enrichment analysis reveals that RNF168 depletion is associated with changes in several pathways, including oestrogen signalling (Figure 3A and Table 2). By overlapping with published ERɑ target genes in MCF‐7 cells with our derived gene expression profiles by RNF168 depletion in the same cell line, 119 ERɑ target genes are significantly decreased, suggesting the regulatory role of RNF168 in ERɑ signalling (Figures 3B and S1). By depletion RNF168 via two different siRNAs, we observe that ERɑ mRNA and protein level are decreased in both MCF‐7 and T47D cells (Figure 3C‐F).

**Table 2:** Changed pathways by RNF168 depletion

Changed pathways by RNF168 depletion	z‐score
ERK_MAPK signaling	−7.04
PAK signaling	−6.95
Cell cycle_G1 or S checkpoint regulation	−5.76
PPAR signaling	−5.15
AMPK signaling	−4.92
Antiproliferative role of somatostatin receptor 2	−4.82
JAK_stat signaling	−4.70
VEGF signaling	−4.47
IL‐1 signaling	−3.87
LXR_RXR activation	−3.86
Oestrogen‐dependent breast cancer signalling	−3.81
FGF signaling	−3.71
Gaq signaling	−3.66
Fc Epsilon Rl signaling	−3.55
HMGB1 signaling	−3.48
Cardiac p‐adrenergic signaling	−3.47
Agrin interactions at neuromuscular junction	−3.42
Androgen signaling	−3.40
HGF signaling	−3.33
CDK5 signaling	−3.28
Tec kinase signaling	−3.22
PI3K.AKT signaling	−3.22
Gas signaling	−3.19
RhoGDI signaling	−3.14
Pancreatic adenocarcinoma siganling	−3.11

### 3.4 RNF168 depletion decreases ERɑ signalling activity in breast cancer cells

**Figure 4:**
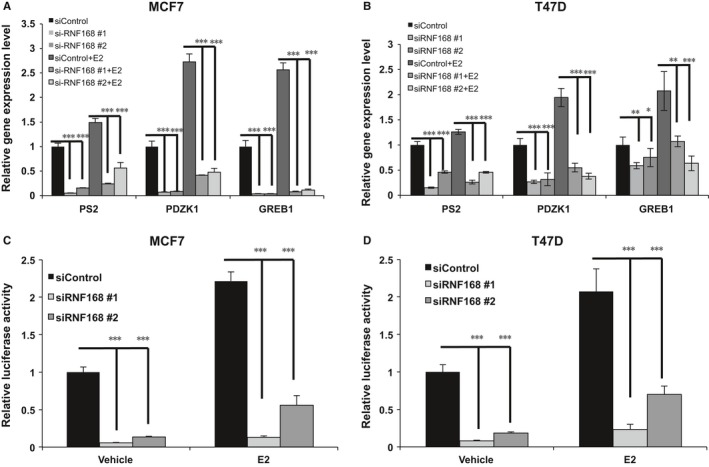
RNF168 depletion decreases ERɑ signalling activity in breast cancer cells. A and B, RNF168 depletion decreases ERα target genes using two different siRNA oligos. MCF‐7 and T47D cells were transfected with siRNF168 or siControl. After 48 h, cells were cultured in phenol red‐free medium and treated with either ethanol or 10 nmol/L estradiol for 6 h. Total RNA was prepared and the expression of the endogenous ERα target genes, PS2, GREB1 and PDZK1 were determined by qPCR. Shown are the results from three experiments. *P < .05; **P < .01; ***P < .001 for target gene expression comparison. C and D, RNF168 depletion affects ERE luciferase activity in MCF7 and T47D cells. MCF7 or T47D cells were transfected with siRNF168 or siControl together with ERE‐luciferase reporter plasmid. Cells were treated with 10 nmol/L estradiol or vehicle. Luciferase activity was measured 48 h after transfection. Shown are the results from three experiments. *P < .05; **P < .01; ***P < .001 for luciferase activity comparison

As RNF168 depletion decreases ERɑ mRNA and protein level, we further assess its impact in ERɑ signalling. Quantitative PCR shows that RNF168 depletion significantly decreases ERɑ classical target gene expression in MCF‐7 and T47D cells, including PS2, GREB1 and PDZK1 (Figures 4A,B and S2A). By measuring ERE (Estrogen Response Element)‐luciferase activity, RNF168 depletion dramatically decreases ER alpha reporter gene activity under both vehicle and estradiol treatment in MCF‐7 and T47D cells (Figure 4C,D). These data indicate that RNF168 is required for ER alpha gene expression and subsequent ERɑ signalling function in breast cancer cells.

### 3.5 Reduction of RNF168 level reduces recruitment of RNF168 to ERɑ promoter—a potential mechanism for ERɑ signalling regulation

**Figure 5:**
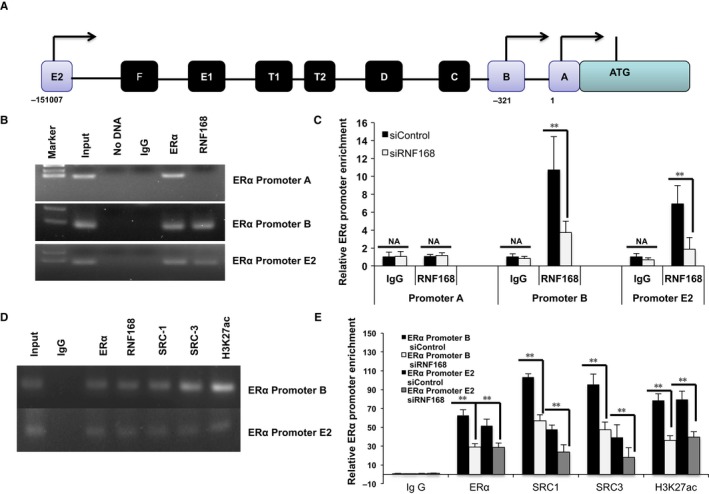
Reduction of RNF168 level reduces recruitment of RNF168 to ERɑ promoter—a potential mechanism for ERɑ signalling regulation. A, Genomic organization of ERα promoter structure of human ERα genes is shown, among which promoter A, promoter B and promoter E2 are used in MCF‐7 cells.^23^ B, ChIP assay shows that RNF168 is recruited to ERα promoter B and E2. MCF7 cells were fixed for 30 min. Rabbit Ig G was used as the negative control, while ERα antibody was used as the positive control. The primer sequences were shown in Table S1. Then enriched DNA fragments were subject to PCR reaction and detected by DNA gel electrophoresis. C, ChIP assay shows that for RNF168 depletion decreases RNF168 recruitment to ERα promoter regions. MCF7 cells were transfected with siRNF168 or siControl for 48 h. After that, cells were fixed for 30 min. Rabbit Ig G was used as the negative control. The primer sequences were shown in Table S1. The relative ERα promoter enrichment was measured by real‐time PCR. *P < .05; **P < .01; ***P < .001 for binding comparison. D, ChIP assay shows that RNF168, ERα, ERα co‐activators (SRC1 and SRC3) and H3K27ac co‐occupy at ERα promoter B and E2. MCF7 cells were fixed for 30 min. Rabbit Ig G was used as the negative control. The primer sequences were shown in Table S1. Then enriched DNA fragments were subject to PCR reaction and detected by DNA gel electrophoresis. E, ChIP assay shows that for RNF168 depletion decreases ERα, ERα co‐activators (SRC1 and SRC3) and H3K27ac recruitment to ERα promoter regions. MCF7 cells were transfected with siRNF168 or siControl for 48 h. After that, cells were fixed for 30 min. Rabbit Ig G was used as the negative control. The primer sequences were shown in Table S1. The relative ERα promoter enrichment was measure by real‐time PCR. *P < .05; **P < .01; ***P < .001 for binding comparison

Further immunoprecipitation (IP) assay is carried out to detect the possible association between RNF168 and ERɑ (Data not shown). The IP assay based on MCF‐7 cell does not indicate the association between RNF168 and ERɑ. As ERɑ mRNA level is also dramatically decreased, we hypothesize that RNF168 might regulate ERɑ transcriptional level. Seven promoters have been identified from ERɑ genes, while only promoter A, B and E2 are utilized for ERɑ expression in MCF‐7 cells (Figure 5A).^23^ Chromatin immunoprecipitation (ChIP) is carried out to detect RNF168 binding to ERɑ promoter regions. As ERɑ has been proved to bind to its own gene promoter regions, ERɑ antibody‐based ChIP is used for positive control. ChIP assay shows that RNF168 could bind to ERɑ promoter B and E2, but not to promoter A, while ERɑ could bind to all the three promoters (Figure 5B). Transfection with siRNA‐targeting RNF168 results in significantly decreased binding at promoter B and E2 (Figure 5C). Further ChIP assay shows RNF168 is recruited together not only ERɑ, but also ERɑ common co‐activators, including SRC1, SRC3 and K27‐linked acetylated form of histone‐3 (H3K27ac) (Figure 5D). Depletion RNF168 dramatically decreases ERɑ, SRC1, SRC3, P300, PolII and H3K27ac binding to ERɑ promoter regions (Figures 5E and S3A), which indicates RNF168 as an important regulator for the formation of transcriptomic complex in regulation ERɑ expression. Coupled with the data that RNF168 depletion significantly decreases ERɑ mRNA level, it indicates that RNF168 binding to ERɑ promoter region could be a potential mechanism in which RNF168 facilitate ERɑ transcription level and ERɑ signalling.

## 4 DISCUSSION

Here, we report that the nuclear E3 ubiquitin ligase RNF168 promotes ERɑ transcription, ERɑ signalling activity and promotes ERɑ‐positive breast cancer cell proliferation. Besides, we also observe the higher expression of RNF168 in breast cancers compared with normal tissue and the poor prognosis survival correlation with RNF168 expression in endocrine therapy patients. Although we fail to detect the direct association between RNF168 and ERɑ, RNF168, which is detected at the ERɑ promoter region, offers a potential mechanism that RNF168 modulates ERɑ signalling via controlling its transcription in breast cancers. On the basis of these data, we propose that selective modulation of RNF168 expression or/and functional cooperation with ERɑ could be a strategy to inhibit ERɑ‐positive breast cancer proliferation.

ERɑ belongs to the nuclear receptor superfamily of transcription factors, and specifically to the ligand‐dependent subfamily.^24^ ERɑ is comprised of three functional domains, including AF1 domain (Activator Function 1 domain), DNA binding domain and AF2 domain (Activator Function 1 domain).^25^ It has been shown that a group of nuclear proteins are involved in regulating ERɑ signalling activity, including co‐activators and corepressors.^26^ However, the detailed mechanism in controlling the transcription level of ERɑ is not totally known. Quite a few studies revealed several transcriptional factors could be recruited to ERɑ promoter regions, such as SP1, AP‐1 and CBP.^27^, ^28^, ^29^ But, recent papers also indicate the important role of histone modification proteins in regulation ERɑ expression.^30^ For example, the histone deacetylase inhibition could rescue ERɑ expression even in ERɑ‐negative breast cancer cells.^31^ In our study, we observed a novel histone modification protein‐RNF168, which modulates ERɑ signalling via direct binding to ERɑ gene promoter regions. However, ERɑ promoter luciferase assay showed RNF168 alone could not induce the luciferase activity (Figure S3B). There are two possible reasons: first, it indicates RNF168 might not regulate ERɑ in a “straight‐forward” manner. Or ERɑ promoter luciferase reporter is a simplified model and could not reflect the gene regulation pattern in the chromatin‐based scale. Our study reveals the possible regulatory role of histone modifiers in regulation ERɑ expression and subsequently ERɑ‐positive cancer biological function. This finding reveals the unconventional function of the chromatin modification proteins in regulation nuclear receptors/transcriptional factors function, such as ERɑ signalling.

Among the 700 putative E3 ubiquitin ligases, the RING protein finger family has attracted recent notice due to the atypical ubiquitination mechanisms and distinct regulatory functions. Among the RING finger protein family,quite a few E3 ligases have been shown to modulate ERɑ signalling via transcriptional or post‐translational regulation of ERɑ.^9^, ^10^, ^12^, ^25^, ^32^ For example, RNF31 and RNF8 were shown to modulate ERɑ protein stability via inducing ERɑ mono‐ubiquitination, while BRCA1 (RNF53) was shown to suppress ERɑ signalling and promote ERɑ degradation.^11^, ^12^, ^32^ However, RBCK1 (RNF54) was shown to modulate ERɑ signalling in two mechanisms. RBCK1 both promotes ERɑ transcription level and also functions as ERɑ co‐activator to promote the transcriptional activity on ERɑ target genes.^10^, ^33^ In our current study, we fail to detect the interaction between RNF168 and ERɑ, which means these two proteins exist in different complexes. However, RNF168 depletion decreases RNF168 binding to ERɑ promoters regions and subsequently shuts down ERɑ transcription and its target genes. Based on these findings, we could conclude RNF168 play an unconventional role in regulating ERɑ signalling and ERɑ‐positive cancer phenotype.

## 5 CONCLUSIONS

Although ERɑ has been well documented to have a critical role in aetiology and progression of breast cancer, RNF168 emerges to be an important component in regulation ERɑ transcription in ERɑ‐positive cancer cells. As modulation of ERɑ levels is one feasible approach to target oestrogen signalling and cell proliferation, RNF168 could be a potential drug target for ERɑ‐positive breast cancers.

## COMPETING INTEREST

The authors declare that they have no competing interests.

## AUTHORS’ CONTRIBUTIONS

TZ, ZHL and HW contributed to the manuscript writing. ZHL, HJY, XL, YXH, YZ, MX, BBW, NY and SFY contributed to the molecular and cellular biology experiments. JHZ, JTX and GU contributed to the clinical data analysis and RNA sequence data analysis. XML, TZ and HW contributed to the funding support for this study.

## AVAILABILITY OF DATA AND MATERIALS

Additional data and materials may be requested from the corresponding author on reasonable request.

## ETHICS APPROVAL AND CONSENT TO PARTICIPATE

Not applicable.

## OPEN ACCESS

This article is distributed under the terms of the Creative Commons Attribution 4.0 International License (http://creativecommons.org/licenses/by/4.0/), which permits unrestricted use, distribution and reproduction in any medium, provided you give appropriate credit to the original author(s) and the source, provide a link to the Creative Commons license and indicate if changes were made. The Creative Commons Public Domain Dedication waiver (http://creativecommons.org/publicdomain/zero/1.0/) applies to the data made available in this article, unless otherwise stated.
