# Supplementary figures and images for: RNF168 facilitates oestrogen receptor ɑ transcription and drives breast cancer proliferation
Source: J Cell Mol Med. 2018 Jul 5;22(9):4161–70. doi: 10.1111/jcmm.13694 (PMC6111850; doi:10.1111/jcmm.13694)

Supplementary Figure 1

A

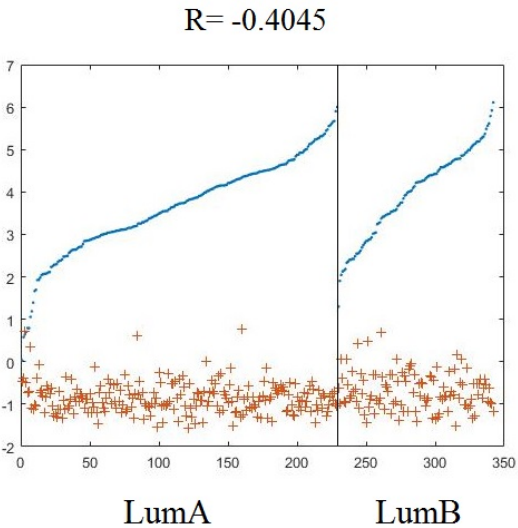

B

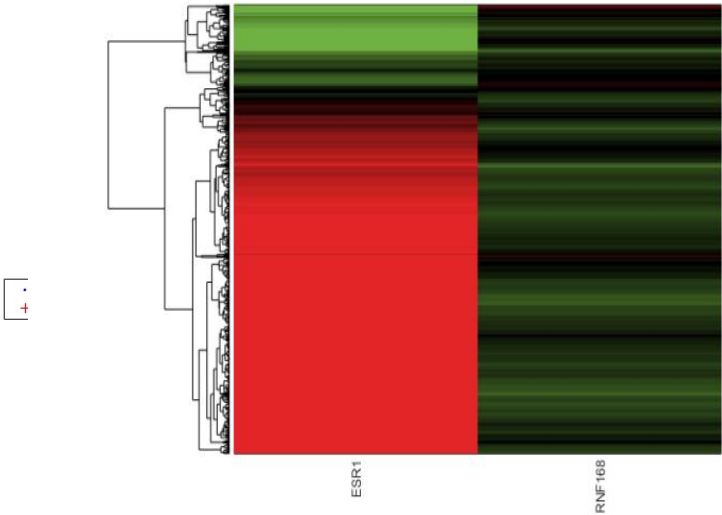

Supplement: Supplementary file 1 [file JCMM-22-4161-s001.pdf]

# Supplementary Figure 2

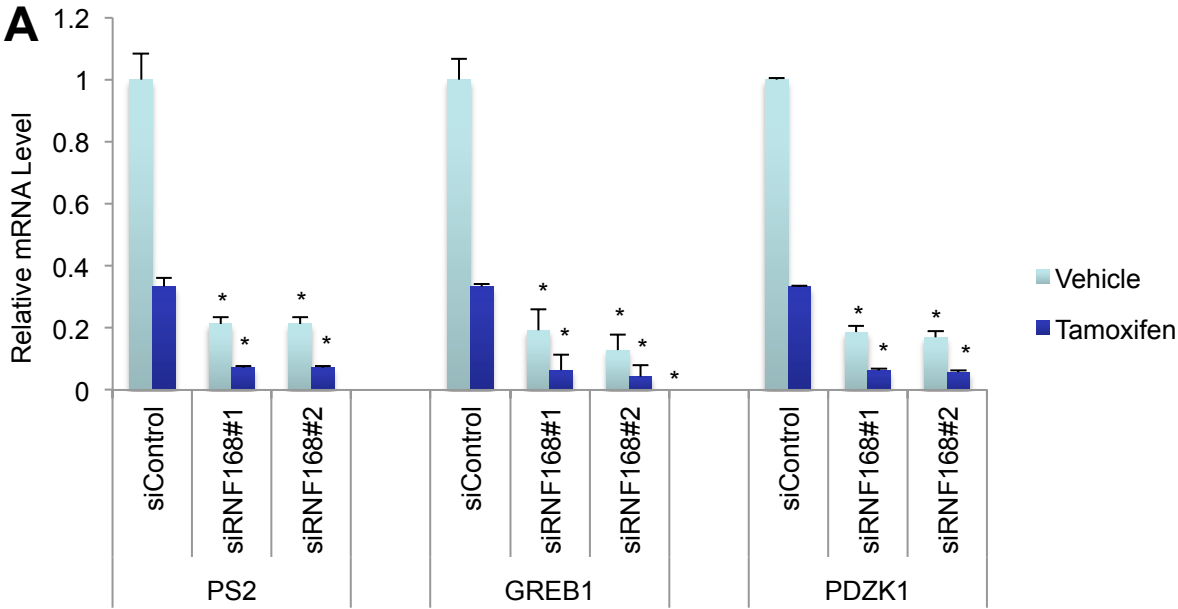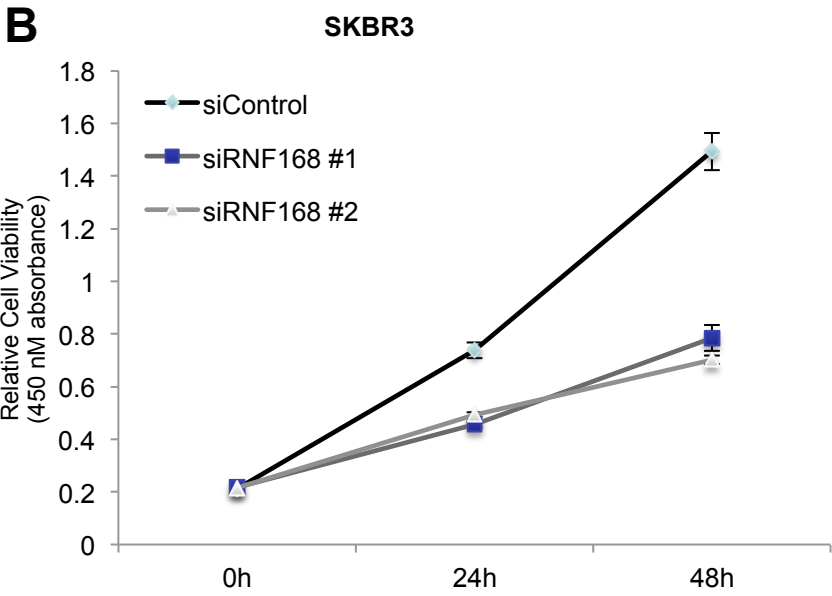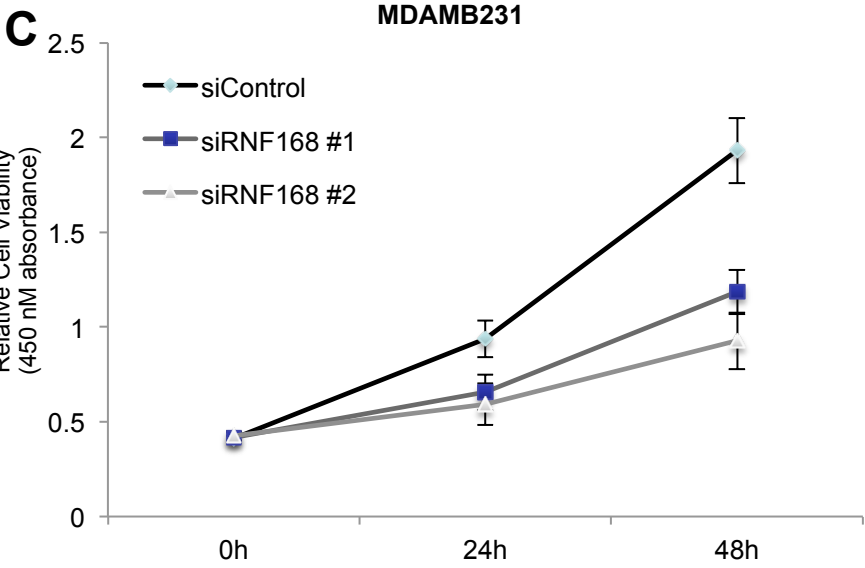

Supplement: Supplementary file 2 [file JCMM-22-4161-s002.pdf]

# Supplementary Figure 3

A

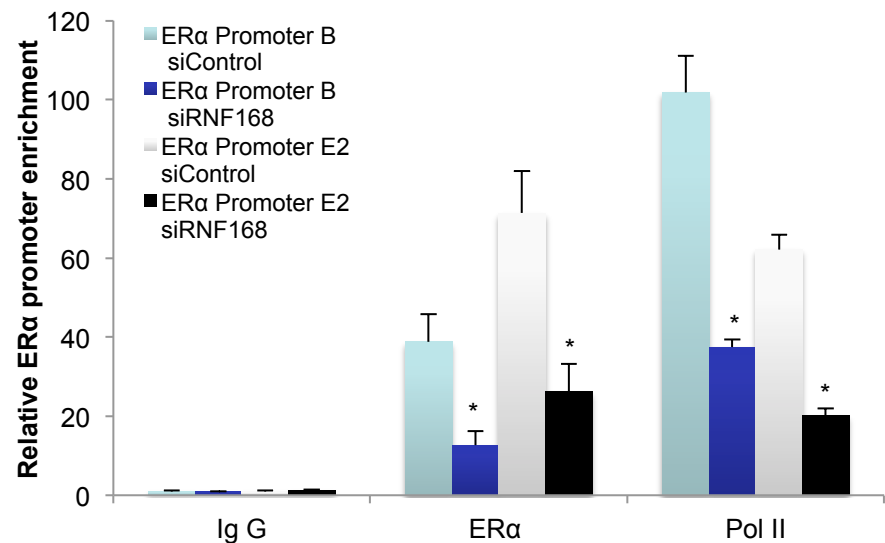

B

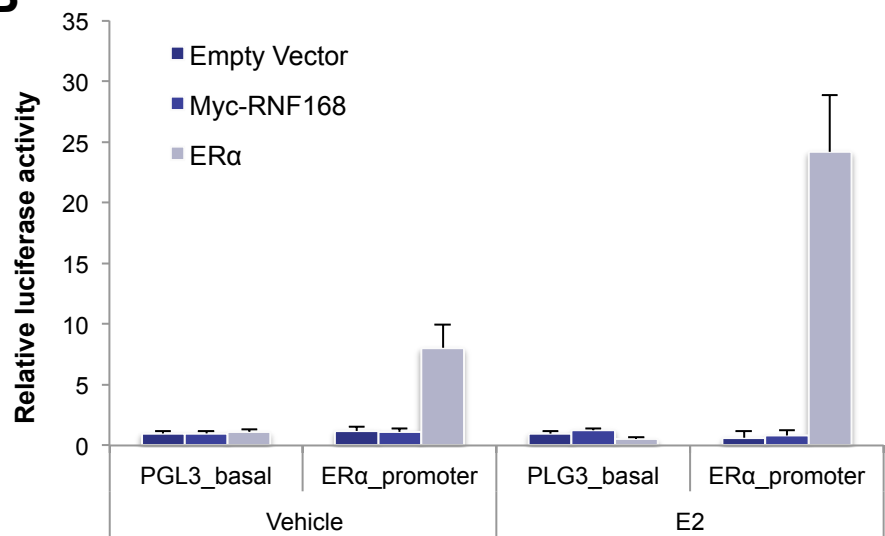

Supplement: Supplementary file 3 [file JCMM-22-4161-s003.pdf]

# Supplementary Figure 4

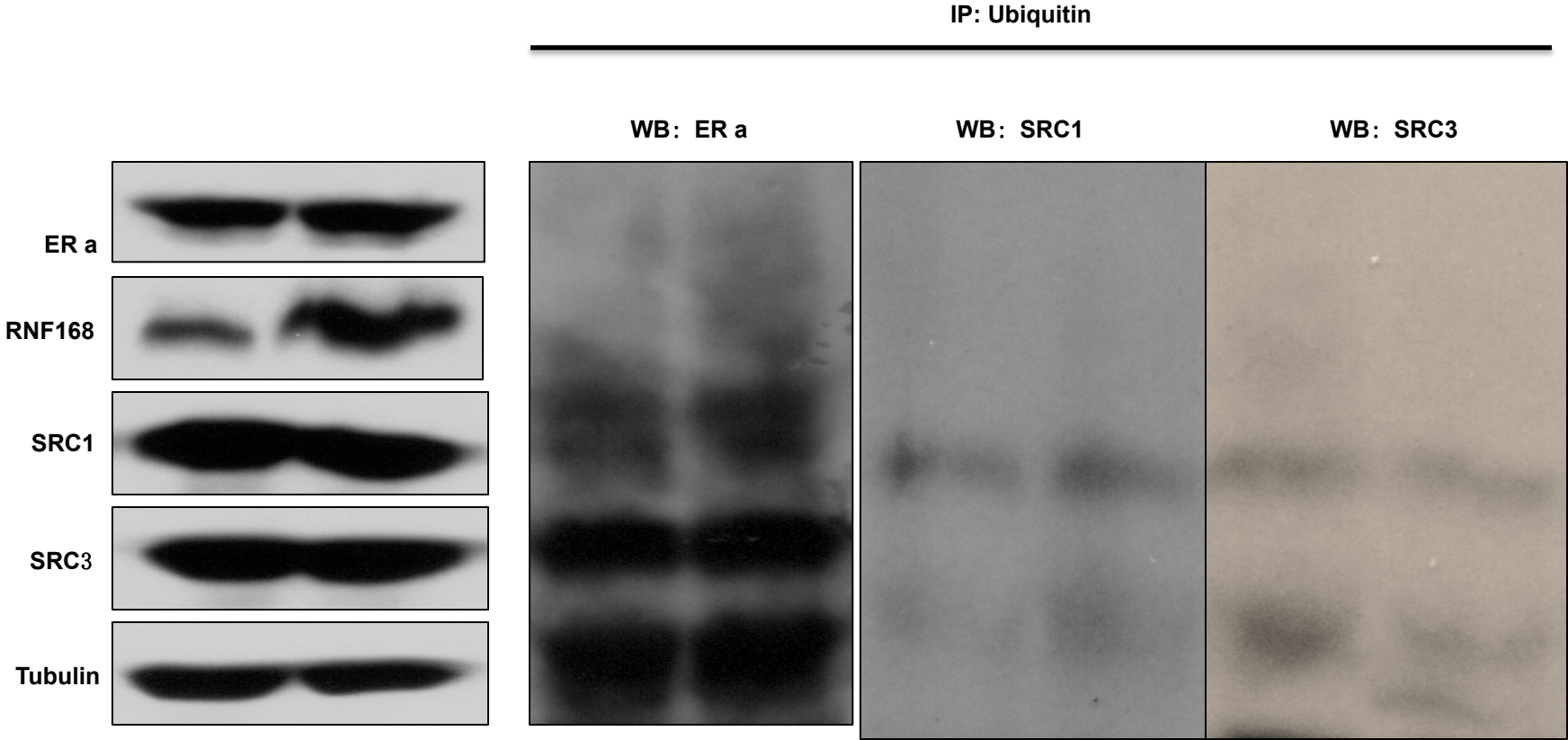

Supplement: Supplementary file 4 [file JCMM-22-4161-s004.pdf]
